# Three-dimensional echocardiography using single-heartbeat modality decreases variability in measuring left ventricular volumes and function in comparison to four-beat technique in atrial fibrillation

**DOI:** 10.1186/1476-7120-8-45

**Published:** 2010-10-05

**Authors:** Kambiz Shahgaldi, Aristomenis Manouras, Anna Abrahamsson, Petri Gudmundsson, Lars-Åke Brodin, Reidar Winter

**Affiliations:** 1 Department of Cardiology, Karolinska University Hospital Huddinge, Stockholm, Sweden; 2 Department of Clinical Physiology, Karolinska University Hospital Huddinge, Stockholm, Sweden; 3 Department of Biomedical Laboratory Science, Malmö University, Malmö, Sweden; 4 School of Technology and Health, Royal Institute of Technology, Huddinge, Sweden

## Abstract

**Background:**

Three dimensional echocardiography (3DE) approaches the accuracy of cardiac magnetic resonance in measuring left ventricular (LV) volumes and ejection fraction (EF). The multibeat modality in comparison to single-beat (SB) requires breath-hold technique and regular heart rhythm which could limit the use of this technique in patients with atrial fibrillation (AF) due to stitching artifact. The study aimed to investigate whether SB full volume 3DE acquisition reduces inter- and intraobserver variability in assessment of LV volumes and EF in comparison to four-beat (4B) ECG-gated full volume 3DE recording in patients with AF.

**Methods:**

A total of 78 patients were included in this study. Fifty-five with sinus rhythm (group A) and 23 having AF (group B). 4B and SB 3DE was performed in all patients. LV volumes and EF was determined by these two modalities and inter- and intraobserver variability was analyzed.

**Results:**

SB modality showed significantly lower inter- and intraobserver variability in group B in comparison to 4B when measuring LV volumes and EF, except for end-systolic volume (ESV) in intraobserver analysis. There were significant differences when calculating the LV volumes (p < 0.001) and EF (p < 0.05) with SB in comparison to 4B in group B.

**Conclusion:**

Single-beat three-dimensional full volume acquisition seems to be superior to four-beat ECG-gated acquisition in measuring left ventricular volumes and ejection fraction in patients having atrial fibrillation. The variability is significantly lower both for ejection fraction and left ventricular volumes.

## Background

Accurate quantification of LV volumes and EF has important diagnostic, prognostic and therapeutic implications. The variety of therapeutic decisions should be on the basis of LV volume and EF measures in various patient groups [1]. 2DE is today the most widely used modality for measuring LVEF, LV end-diastolic volume (EDV) and LVESV. However, 3DE is increasingly available, and several reports have demonstrated the superiority of 3DE regarding LV volume and EF measurements [2-9].

The most commonly used 3DE method for volume and EF measurement is to use real-time ECG- gated volume stitching from four consecutive 4B with the purpose to maintain an acceptable spatial and temporal resolution [7]. The recently developed SB method has some potential advantages, despite suffering from some degree of decreased spatial and temporal resolution. This modality will further advance the assessment of LV by improving the speed of acquisition and reducing stitching artifacts. This is especially true for patient in AF.

Assessment of LVEF during AF has conventionally proved difficult because of beat-to-beat variation [10-13]. Due to the variability, the standard protocol for obtaining an accurate assessment of LV function during AF involves averaging a random number of consecutive cardiac cycles. The result is usually unreliable because the averaged value is dependent on a selected window of cardiac cycles and the mean number of cardiac cycles required in AF is approximately 3 times that required in sinus rhythm (SR) [14]. It is time-consuming and not realistic in the clinical scenario to analyze more than 10 beats for evaluating LV performance. It is well known that LVEF during AF varies depending on the preceding cardiac cycle length [15,16]. In clinical practice in AF patients, LVEF is commonly measured from a single beat either using a visual assessment for targeting a specific heart beat having a visually assessed representative EF, or looking for an average R-R interval for the representative heart beat to measure from. Thus in AF patients particularly, 3DE becomes impractical for these reasons. However, SB could have an advantage over 4B since it is possible to choose a representative heart beat similarly to 2DE, and furthermore due to the lack of stitching artifact.

The study sought to investigate whether SB full volume 3DE acquisition reduces inter- and intraobserver variability when measuring LV volumes and EF in comparison to 4B ECG-gated full volume 3DE recording in patients with AF.

## Method

We included fifty-five consecutive adult patients (37 men and 18 women, aged 53 ± 17 years of age, group A) having SR (67 ± 10 beats/sec) who were referred to the echocardiographic examinations on varying clinical reasons (Table 1) at the Department of Cardiology, Karolinska university Hospital, Huddinge. We also included twenty-three patients (12 men and 11 women, aged 65 ± 12 years of age, group B) having AF (97 ± 27 beats/sec). Contrast agents were not used in this study. The study protocol was approved by the ethics committee of Karolinska University Hospital, Stockholm, Sweden, and all patients gave informed consent. Clinical characteristics of the study population are displayed in Table 1.

**Table 1:** Clinical characteristics of the study population.

	Group A	Group B
Age (years)	53 ± 17	65 ± 12
Males (%)	67	52
Heart rate (bpm)	67 ± 10	97 ± 27
**Indication of echo study (%)**		
Suspected heart failure	60	29
Murmur/Valvular heart disease	15	23
Suspicion of Left ventricular thrombus	5	8
Routine control of transplanted heart patients	10	0
Unspecific exclusion of cardiac pathology	0	20
Other	10	20

A complete 2DE and Doppler study was performed in all patients, using a commercially available Vivid E9 ultrasound machine (GE Healthcare, Horten, Norway) equipped with M5S probe. All acquisitions were performed by the same experienced operator with the patients in the left lateral position. Data sets were stored digitally for off-line analysis using commercially available software (EchoPAC PC version 108.1.4, GE Healthcare).

A novel 3V matrix-array transducer was used for acquiring the 3D images. Gain and compression controls, as well as time gain compensation settings were optimized to enhance image quality.

**Figure 1:**
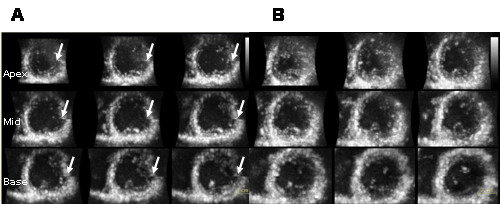
**(A) Four-beat ECG-gated full volume three-dimensional loop presented in 9-slice of the left ventricular (from apex to base)**. Note the stitching artifacts due to irregular heart rate in all 9-slices in an atrial fibrillation patient (marked with arrows). (**B**) Single-beat three dimensional echocardiography presented in 9-slice. No stitching artifacts can be detected in a patient having atrial fibrillation.

**Figure 2:**
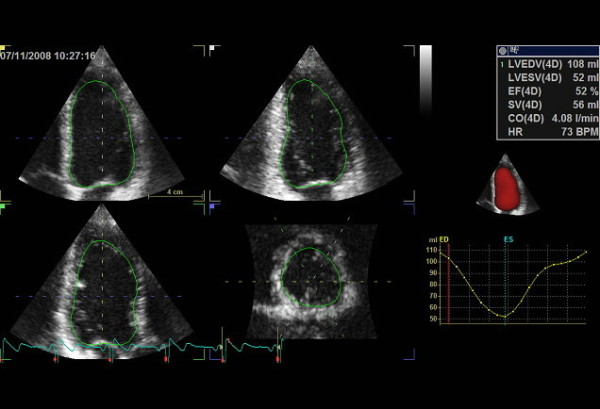
**Quad view presentation of left ventricular using 4D auto LVQ software for measurement of left ventricular volumes and ejection fraction with three-dimensional echocardiography**. Volume time-plot and quantitative analysis and three-dimensional model are presented in the right panel.

A full volume scan was acquired from four consecutive cardiac cycles (fig. 1A) and 1 cardiac cycle, single-beat (fig. 1B) immediately after each other for each patient during end expiration breath-hold. Volume analysis was made using a commercially available semi-automated analysis tool, 4D auto LV volume quantification (4DLVQ, EchoPAC PC version 108.1.4, GE Healthcare) which has previously been validated [17,18]. The ED frames for contour detection were automatically displayed in quad-view: apical four-, two-chamber, three-chamber and LV short-axis plane (fig. 2). Manual positioning by translating the four-chamber plane was first performed in order that the corresponding intersection line of all planes was placed in the middle of LV cavity, crossing the LV apex and the centre of mitral valve opening in each view. The software required manual input of three points for each of the three apical planes (two points at mitral annulus borders, and one at the apex) first in ED frames, and then continuing for ES frames. The software automatically delineates the LV endocardial border in a 3D-model from ED and ES phases. In cases where the automatic delineation of the endocardial border was considered suboptimal the borders could be adjusted manually. LVEDV, LVESV and EF were finally displayed.

Two readers analyzed the 3D images twice blinded to all clinical data and previous reading. All measurements were made twice one week apart.

## Statistical analysis

All data are expressed as mean ± SD. Bland and Altman analysis was performed to determine the systematic bias and limits of agreement of LV volumes and EF between the different methods [19]. In order to determine reproducibility all measurements were analyzed twice with one week apart blinded to the results of the first observer. The inter- and intraobserver variability was measured according to the following formula: (SD_diff _× 100%)/total mean × √2 (Dahlberg's formula) [20], where SD_diff _is the SD of difference between measurements. All variables were tested for normality using the Kolmogorov-Smirnov test. Different echocardiographic data were tested using paired t-tests. The significance level was set as p < 0.05. Statistical analysis was performed using standard statistical software (SPSS version 16.0, Inc, Chicago, IL).

## Results

Echocardiographic analyses were successfully completed in seventy patients. Five patients in group A and three patients in group B were excluded from the study due to poor acoustic window or because clear endocardial border visualization was difficult (two segments or more). Average image time resolution for 4B was 72 ± 18 volumes per second, whereas for SB data sets were acquired at 21 ± 5 volumes per second.

The mean EDV, ESV and EF in group A using 4B and SB is presented in table 2. Statistical analysis showed no significant difference between the variables using 4B in comparison to SB.

**Table 2:** Mean ± SD values for left ventricular end-diastolic volume (ml), end-systolic volume (ml) and ejection fraction (%) for the different imaging strategies

	EDV (ml)	ESV (ml)	EF (%)	HR (bpm)
**Group A**				
Four-beat full volume	102 ± 25	47 ± 19	54 ± 10	67 ± 10
Single-beat full volume	99 ± 25	47 ± 20	53 ± 10	67 ± 11
				
**Group B**				
Four-beat full volume	87 ± 27***	52 ± 22***	41 ± 11*	97 ± 27
Single-beat full volume	119 ± 34	64 ± 27	47 ± 12	97 ± 26

**EDV: **end-diastolic volume, **ESV: **end-systolic volume, **EF: **ejection fraction, **HR: **heart rate,

*** p < 0,0001 vs. end-diastolic, end-systolic volume and *p < 0.05 vs. ejection fraction by single beat.

The mean EDV, ESV and EF by 4B and SB in group B are demonstrated in table 2. Statistical analysis showed significant differences between EDV, ESV (p < 0.001) and EF (p < 0.05) by 4B in comparison to SB.

**Figure 3:**
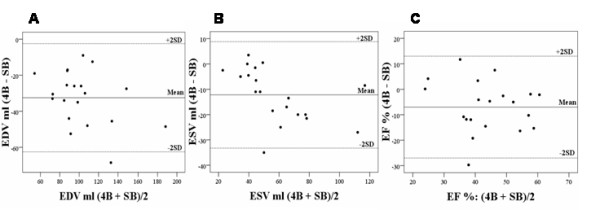
**A-C**. Bland-Altman plot of differences between end-diastolic volume (**A**), end-systolic volume (**B**) and ejection fraction (**C**) determined by four-beat and and single-beat three-dimensional echocardiography in atrial fibrillation patients.

The limits of agreement analysis of LV volumes and EF in group B by different methods are shown in table 3. In group B there was a mean difference of -32.5 ± 15 ml for EDV by the two different techniques and -12.2 ± 10.5 ml for ESV and -7 ± 10% for EF (fig. 3A,B and 3C).

**Table 3:** Mean differences (ml) and limits of agreement (mean ± SD) in left ventricular volumes and ejection fraction measured by single beat and four-beat ECG-gated three-dimensional echocardiography in patients with atrial fibrillation

	Group B	Group B	Group B
	EDV 4B - EDV SB	ESV 4B - ESV SB	EF 4B - EF SB
Mean difference ± SD	-32.5 ± 15	-12.2 ± 10.5	-7 ± 10
Limits of agreement	-62.5 to -2.5	-33.2 to 8.8	-27 to 13

**EDV: **end-diastolic volume, **ESV: **end-systolic volume, **EF: **ejection fraction,

**4B: **four-beat, **SB: **single-beat.

### Reproducibility

The total patient population both in group A and B were analyzed to determine interobserver reproducibility. Intra- and interobserver variability in group A and B using 4B and SB is presented in table 4. Statistical analysis showed no significant differences between the intra- and interobserver variability in group A. Although, there were significant differences in inter- and intraobserver variability in all three variables in group B except ESV analyzed by intraobserver variability.

**Table 4:** Intra- and interobserver reproducibilities for left ventricular volumes and ejection fraction by single- and four-beat  three-dimensional echocardiography.

	EDV (ml)	ESV (ml)	EF (%)
**Group A**			
Intraobserver variability (%)			
Four-beat full volume	4.4	5.1	3.4
Single-beat full volume	3.9	4.6	4.1
			
**Group A**			
Interobserver variability (%)			
Four-beat full volume	7.3	8.5	3.9
Single-beat full volume	6.4	6.5	4.5
			
**Group B**			
Intraobserver variability (%)			
Four-beat full volume	9*	11.4	8.3**
Single-beat full volume	4.5	2.9	4.8
			
**Group B**			
Interobserver variability (%)			
Four-beat full volume	10.4*	15.2*	17.9**
Single-beat full volume	7.6	7.2	5.6

**EDV: **end-diastolic volume, **ESV: **end-systolic volume and **EF: **ejection fraction, *p < 0.05 and **p < 0.001.

There were no significant differences in heart rate during acquisition period by 4B and SB modality.

## Discussion

To our knowledge, this is the first study comparing two 3DE techniques side-by-side, 4B and SB in AF. Currently, quantitative measurements of LV size and function are most commonly obtained using the biplane Simpson's rule [21] with 2D transthoracic echocardiography, which is highly dependent on operator technique and which can be limited by poor acoustic windows and the need for geometric assumptions. Volumetric methods of image acquisition by 3DE have demonstrated substantial improvements in accuracy and reproducibility over 2DE [22,23].

In this study, LV volumes and EF in group A were comparable in these two different acquisition techniques (4B and SB respectively) without any significant differences. This finding is not in concordance by a recent publication by Macron L et al. [24]. The only explanation is that we had higher time resolution using the SB modality which prevented over/underestimation of LV volumes. SB showed a tendency to a lower inter- and intraobserver variability when measuring LV volumes in comparison to the 4B technique in group A, although, this was not statistically significant. Previous studies have shown the low inter- and intraobserver variablity of LV volumes and EF measurements using 3DE in patient with SR [25-27]. To our knowledge, there are no studies investigating variability of measuring LV volume and EF in AF patients using 3DE. In our study we demonstrated significantly lower intra- and interobserver variability of LV volumes and EF using SB in comparison to 4B in patients having AF, except for ESV analysed by intraobserver variability measurement.

EF measurements in AF patients are challenging in clinically practice due to the need of measuring an average of heart beats [14]. From a practical point of view, experienced echocardiographer can overcome this difficulty by selecting visually estimated representative heart beat for EF measurement. Thus in AF patients particularly, 4B becomes impractical for these reasons. However, SB could have an advantage over 4B since it is possible to choose a representative heart beat similarly to 2DE, and furthermore due to the lack of stitching artifacts.

In group B we found significant differences in EDV, ESV and EF when comparing 4B vs. SB which was not surprising considering to beat-to-beat variation. The mean differences were rather poor, -32.5 ± 15 ml for EDV, -12.2 ± 10.5 ml for ESV and -7 ± 10% for EF between 4B and SB, especially considering that this patient population had normal LV volumes. Additionally, CI for the respective measurements was rather wide, 65 ml, 42 ml and 40% respectively. Again, this is expected in AF patients and this study was not addressed to investigate this issue. Furthermore, LV volumes and EF using 4B were significantly smaller in comparison to SB (p < 0.0001 for LV-volumes and p < 0.05 for EF). From clinical point of view, perhaps the most important finding in this study is the lower variability of SB in comparison to 4B when measuring LV volumes and EF in AF (e.g. 5.6% vs. 17.9% for EF determination, interobserver variability, p < 0.001). One explanation might be that the software has difficulties in tracking the endocardial border in AF when gathering volume in 4B because of stitching artifacts due to the irregular heart rate (fig. 1A). The geometrical model in 4D LVQ is flexible and allows a wide variety of shapes. Although, the software has difficulties in achieving the accurate balance between smooth surfaces and surfaces that are improbable [28]. Another explanation could be that prolonged acquisition time using 4B increase the chance of patient motion or artifatcs, resulting in unsuccesfull 3D image reconstruction, which can be overcome using SB [29]. There is a significant user dependency in image quality in echocardiography, i.e. small changes in manual optimization can have a large impact in image quality. The beat-to-beat variability might create a poorer image quality from cardiac movement throughout the 4B acquisition. Interestingly, the LV volumes and EF is significantlly smaller with 4B in comparison to SB. This finding is difficult to explain from any other source than software algorithm, since the measured heart beat was not deliberately selected. In other words, there is no reason to believe that there would be any differences in LV volumes between the methods. The only two possible explanations are either random findings or more probable systematic software error. Providing that this is true, there should be additional advantage in measuring more true volumes when using SB acquisition in patients with irregular heart rhythm.

## Limitations

We have only analyzed our data using the GE equipment/software and this is likely that the effect might depend upon the software and equipment being used.

There are today three software packages from three different vendor's commercially available for the measurement of 3D data. These softwares work with different algorithm and are not automatically interchangeable. To avoid future problems with vendor's specific results this should be tested separately or ideally it would desirable to use same algorithms.

We have not been able to measure the R-R interval in the each three-dimensional modality since this was not possible using the current software. A more robust test-retest variablity would perhaps been if the patients were rescanned and separate datasets acquired and analysed. This might be a true analysis of test-retest variability and would be more clinically relevant.

### Clinical implications

SB modality is a relatively new 3DE technique which reduces intra- and interobserver variability in patients with irregular heart rhythm. SB leads to loss of stitching artifact and reduces the acquisition time. Therefore, when 3DE is used in clinical practice in AF patients, SB modality should be recommended before multi-beat technique.

## Conclusion

Single-beat three-dimensional full volume acquisition seems to be superior to four-beat ECG -gated acquisition in measuring LV volumes and EF in patients having atrial fibrillation. The variability is significantly lower both for EF and LV volumes. More studies are needed to confirm this before implementing single-beat in every day clinical practice.

## Competing interests

The authors declare that they have no competing interests.

## Authors' contributions

RW and KS introduced the study idea. KS acquired the ultrasound images. AA and KS performed the off-line analysis. AM helped in the interpretation of the results and statistical analysis. KS wrote the manuscript, RW and PG added clinical discussion to the manuscript. LÅB reviewed the manuscript. Finally, all authors read and approved the manuscript.
